# One-Class Support Vector Machines Identify the Language and Default Mode Regions As Common Patterns of Structural Alterations in Young Children with Autism Spectrum Disorders

**DOI:** 10.3389/fnins.2016.00306

**Published:** 2016-06-29

**Authors:** Alessandra Retico, Ilaria Gori, Alessia Giuliano, Filippo Muratori, Sara Calderoni

**Affiliations:** ^1^Pisa Division, National Institute for Nuclear PhysicsPisa, Italy; ^2^Department of Chemistry and Pharmacy, University of SassariSassari, Italy; ^3^Department of Physics, University of PisaPisa, Italy; ^4^Department of Developmental Neuroscience, IRCCS Stella Maris FoundationPisa, Italy; ^5^Department of Clinical and Experimental Medicine, University of PisaPisa, Italy

**Keywords:** features classification, One-class support vector machine, Brain Magnetic Resonance Imaging (MRI), autism spectrum disorders, preschool children

## Abstract

The identification of reliable brain endophenotypes of autism spectrum disorders (ASD) has been hampered to date by the heterogeneity in the neuroanatomical abnormalities detected in this condition. To handle the complexity of neuroimaging data and to convert brain images in informative biomarkers of pathology, multivariate analysis techniques based on Support Vector Machines (SVM) have been widely used in several disease conditions. They are usually trained to distinguish patients from healthy control subjects by making a binary classification. Here, we propose the use of the One-Class Classification (OCC) or Data Description method that, in contrast to two-class classification, is based on a description of one class of objects only. This approach, by defining a multivariate normative rule on one class of subjects, allows recognizing examples from a different category as outliers. We applied the OCC to 314 regional features extracted from brain structural Magnetic Resonance Imaging (MRI) scans of young children with ASD (21 males and 20 females) and control subjects (20 males and 20 females), matched on age [range: 22–72 months of age; mean = 49 months] and non-verbal intelligence quotient (NVIQ) [range: 31–123; mean = 73]. We demonstrated that a common pattern of features characterize the ASD population. The OCC SVM trained on the group of ASD subjects showed the following performances in the ASD vs. controls separation: the area under the receiver operating characteristic curve (AUC) was 0.74 for the male and 0.68 for the female population, respectively. Notably, the ASD vs. controls discrimination results were maximized when evaluated on the subsamples of subjects with NVIQ ≥ 70, leading to AUC = 0.81 for the male and AUC = 0.72 for the female populations, respectively. Language regions and regions from the default mode network—posterior cingulate cortex, pars opercularis and pars triangularis of the inferior frontal gyrus, and transverse temporal gyrus—contributed most to distinguishing individuals with ASD from controls, arguing for the crucial role of these areas in the ASD pathophysiology. The observed brain patterns associate preschoolers with ASD independently of their age, gender and NVIQ and therefore they are expected to constitute part of the ASD brain endophenotype.

## Introduction

Different approaches have been proposed to date to explore the clinical (Grzadzinski et al., 2013), genetic (De Rubeis and Buxbaum, 2015), and neurobiological (Hernandez et al., 2015) heterogeneity of Autism Spectrum Disorders (ASD), which are complex neurodevelopmental conditions affecting 1 in 68 children in USA (Centers for Disease Control Prevention, 2014), and are characterized by impairment in socio-communicative abilities, as well as restricted and stereotyped behaviors (American Psychiatric Association, 2013). The non-invasive and non-harmful Magnetic Resonance Imaging (MRI) is a promising tool to study and characterize ASD, as it allows the *in vivo* observation of the brain involvement in the disorder. Several post-processing methods to analyze brain MRI data were developed and a wide range of studies aimed to explore the predictive power of MRI by comparing the brain characteristics of patients with ASD and controls with the final aim of identifying reliable markers of ASD diagnosis (Ecker et al., 2010a,b; Jiao et al., 2010; Ingalhalikar et al., 2011; Calderoni et al., 2012; Wee et al., 2014; Zhou et al., 2014; Gori et al., 2015; Retico et al., 2016).

Machine-learning techniques, e.g., those based on support vector machines (SVMs; Vapnik, 1995), have been shown to be valuable tools to make predictive diagnoses in single subjects in a large variety of psychiatric and neurodevelopmental disorders (Wolfers et al., 2015), including ASD (Retico et al., 2014). They can be implemented for diagnosis prediction, for assessment of the disease progression and to evaluate the treatment effectiveness (Orrù et al., 2012). Machine learning refers to all procedures where the learning by example paradigm is implemented. In most cases conventional binary (also called two-class) classification algorithms are applied to image features to classify an unknown object into one of two pre-defined categories. The classification is particularly challenging when dealing with psychiatric disorders, as the reported neuroanatomical alterations are often very small and quite un-replicated among different studies. Subtle signs of pathology are difficult to catch especially in extremely heterogeneous conditions such as ASD.

In the present study, we propose the use of the One-Class Classification (OCC) or Data Description method (Moya et al., 1993), which, in contrast to two-class classification, makes a description of a single class of objects only (referred to as the positive class or target class) and detects which (new) objects resemble this target class, thus distinguishing them from examples considered outliers. Using OCC instead of two-class classification methods in standard binary classification problems, where objects from both the classes are at disposal, could result in worse recognition accuracy, as the complete knowledge encoded in the available training set is not fully exploited. However, OCC could provide more robustness in case of difficulties embedded in the nature of data, since they seek to describe properties of the target class instead of minimizing the classification error. Therefore, in case of difficult datasets (e.g., when the positive class is well-characterized, whereas the negative class is not sufficiently representative of the negative population) it could be useful to transform the binary classification problem into an OCC task.

The usefulness of OCC in the biomedical domain was already proved in a number of applications, e.g., the automatic recognition of the hypertension type (Krawczyk and Woźniak, 2015) or breast cancer biopsy and 3D optical coherence tomography (OCT) retinal image classification (Zhang et al., 2014) and on brain MRI data. In the latter domain OCC were implemented to learn multivariate normative rules on a group of healthy individuals, thus allowing the interpretation of the distance to the OCC boundary as an abnormality score (Mourão-Miranda et al., 2011; Sato et al., 2012a,b). Working on both voxel- and region-based features, Mourão-Miranda et al. (2011) investigated whether patterns of fMRI response to sad facial expressions in depressed patients would be classified as outliers in relation to patterns of healthy control subjects. They interestingly found out that most patients classified as non-outliers responded to treatments and most patients classified as outliers did not respond to treatments. Sato et al. (2012b) obtained an OCC-based abnormality index analyzing functional connectivity patterns of adults and children with Attention Deficit and Hyperactivity Disorder (ADHD), and found out that the ADHD patients differ significantly from the age-matched control group, showing instead stronger similarity to the group of younger control subjects.

We analyzed with OCC the features extracted from brain structural MRI data in order to measure the OCC performance in the discrimination of patients with ASD with respect to controls in the preschool age. Moreover, we used the OCC to define multivariate normative rules, i.e., we investigated the distribution of patterns of brain structures in control subjects to test the homogeneity of the latter sample and its potential to enable the definition of a robust boundary in relation to which the patients with ASD would be classified as outliers. To carry out a symmetry test, we investigated also whether a consistent neuroanatomical pattern among the ASD patients allows the definition of a robust boundary in relation to which the controls are classified as outliers.

Finally, the relative contribution of the anatomical brain features to the decision function was studied to localize the regions more involved in the one-class classifier boundary definition. To this purpose we extended to the case of OCC the previous literature referring to the generation of a *preimage* when non-linear kernel SVM are used to localize the relevant brain features (Schölkopf et al., 1999). Moreover, the permutation testing usually implemented in two-class classification problems (Mourão-Miranda et al., 2005; Wanh et al., 2007; Gaonkar and Davatzikos, 2013; Gori et al., 2015) was extended to the OCC formulation to allow assigning a statistical significance both to the classification performances and to the brain features contributing most to the OCC boundary definition.

## Materials and methods

### Participants and MRI data acquisition

A group of 21 male and 20 female preschoolers with ASD [mean age ± standard deviation (*SD*) = 49 ± 12 months; age range = 28–70 months] and a group of 40 control subjects matched by gender, age, non-verbal IQ (NVIQ), and socioeconomic status were selected for this case-control study (see Table 1).

**Table 1:** **Dataset composition and sample characteristics**.

**Variable**	**Subject group, mean** ± **std [range]**							
	**ASD (*****n*** = **41)**				**Controls (*****n*** = **40)**			
Age (months)	49 ± 12 [28–70]				49± 14 [22–72]			
NVIQ	73 ± 22 [34–113]				73 ± 23 [31–123]			
	**Males (*****n*** = **21)**		**Females (*****n*** = **20)**		**Males (*****n*** = **20)**		**Females (*****n*** = **20)**	
Age (months)	50 ± 10 [34–70]		48 ± 13 [28 – 69]		48 ± 13 [24–70]		50 ± 16 [22–72]	
NVIQ	75 ± 22 [40–113]		70 ± 23 [34–113]		73 ± 23 [32–123]		72 ± 24 [31–106]	
	**DD (*****n*** = **9)**	**no-DD (*****n*** = **12)**	**DD (*****n*** = **10)**	**no-DD (*****n*** = **10)**	**DD (*****n*** = **10)**	**no-DD (*****n*** = **10)**	**DD (*****n*** = **10)**	**no-DD (*****n*** = **10)**
Age (months)	52 ± 11 [37–70]	48 ± 9 [34–66]	43 ± 14 [28–69]	54 ± 10 [36–69]	52 ± 13 [24–70]	45 ± 13 [30–65]	51 ± 14 [30–66]	50 ± 18 [22–72]
NVIQ	54 ± 8 [40–66]	91 ± 14 [70–113]	51 ± 10 [34–65]	89 ± 14 [73–113]	54 ± 11 [32–68]	92 ± 15 [74–123]	52 ± 13 [31–68]	93 ± 10 [73–106]

*ASD, autism spectrum disorders; NVIQ, non-verbal intelligence quotient; std, standard deviation; DD, developmental delay (NVIQ < 70); no-DD, without developmental delay (NVIQ ≥ 70)*.

Participants in the ASD group were recruited at the ASD Unit of IRCCS Stella Maris Foundation (Pisa), a tertiary hospital and research university in Italy. They were rigorously diagnosed with ASD according to the DSM-IV-TR criteria (American Psychiatric Association, 2000) by a multidisciplinary team including a senior child psychiatrist, an experienced clinical child psychologist and a speech–language pathologist during 3–5 days of extensive evaluation, and confirmed by the ADOS-G (Lord et al., 2000) administrated by clinical psychologists who met standard requirements for research reliability. ASD patients were included if their age was between 2 and 6 years and their NVIQ ≥ 30. Exclusion criteria were: (a) anomalies detected by MRI (b) neurological syndromes or focal neurological signs; (c) significant sensory impairment (e.g., blindness, deafness); (d) anamnesis of birth asphyxia, premature birth, or epilepsy; (e) use of any psychotropic medication; (f) absence of major dysmorphic features including microcephaly or macrocephaly; and (g) potential secondary causes of ASD determined by high-resolution karyotyping, DNA analysis of Fragile-X, or screening tests for inborn errors of metabolism (plasma and urine aminoacid analysis, urine organic acid measurement, urine mucopolysaccarides quantitation, plasma and urine creatine, and guanidinoacetate analysis). The control group was composed of 20 preschoolers with idiopathic developmental delay (DD), i.e., with NVIQ score < 70, and 20 preschoolers without developmental delay (noDD), i.e., with NVIQ score ≥ 70, recruited at the same hospital. Subjects with DD were included within the control group in order to obtain a match for NVIQ between patients and controls, as well as to increase the size of the data sample under investigation. The diagnosis of idiopathic DD was made after a negative thorough assessment for underlying causes, including audiometry, thyroid hormone disorders, high-resolution karyotyping, DNA analysis of Fragile-X and screening tests for inborn errors of metabolism. The control group was selected so as to meet the same exclusionary criteria as the ASD patients—except the criterion (g) specified above—with the further requirements of exclusion of ASD diagnosis (performed by a senior child psychiatrist and based on the DSM-IV-TR criteria), and no family history of ASD. ASD and DD patients underwent the brain MRI examination as a completion of the assessment pathway with the aim of excluding brain alteration, whereas noDD subjects performed brain MRI because of various reasons (including headache, seizures with fever, strabismus, cataract, paroxysmal vertigo, diplopia).

MRI data were acquired using a GE 1.5 T Signa Neuro-optimized System (General Electric Medical Systems) at IRCCS Stella Maris Foundation, fitted with 40 mT/m high-speed gradients. Within the MRI protocol for children a whole-brain fast spoiled gradient recalled acquisition in the steady-state T1-weighted series (FSPGR) was collected in the axial plane with repetition time 12.4 ms, echo time 2.4 ms, inversion time 700 ms, flip angle of 10°, yielding to contiguous axial slices with voxel size of 1.1 × 1.1 × 1.1 mm^3^. All children were sedated with a general anesthesia with a halogenated agent while spontaneously breathing. For all MRIs performed between September 2006 and February 2013 the same sequence of acquisition was used and the written informed consent from a parent or guardian of children was obtained. The research protocol was approved by the Institutional Review Board of the Clinical Research Institute for Child and Adolescent Neurology and Psychiatry.

### Data preprocessing and feature extraction

The preprocessing of the entire data set of structural MRI included the volumetric segmentation and cortical reconstruction by the Freesurfer image analysis suite version 5.1.0, documented and freely available online (http://freesurfer.net/; Fischl et al., 1999, 2004; Klein and Tourville, 2012). In the cortical parcellation step, Freesurfer assigns a neuroanatomical label to each location on the cortical surface according to a previously prepared atlas file. We used the Desikan-Killiany-Tourville (DKT) cortical atlas which divides the cerebral cortex into 62 structures (31 structures per hemisphere) (Klein and Tourville, 2012): 14 in the temporal lobe, 20 in the frontal lobe, 10 in the parietal lobe, 8 in the occipital lobe, and 10 in the cingulate cortex.

The following five surface-based features for each of the 62 DTK structures are computed: *Area* (white surface area in mm^2^); *Volume* (gray matter volume in mm^3^); *Thickness* (average cortical thickness in mm); *ThicknessStd* (standard deviation of cortical thickness in mm); *Mean-Curv* (integrated rectified mean curvature in mm^−1^). The *Volume* is computed according to a surface-based method, as the average of the white and pial surface areas, multiplied by the cortical thickness. In addition we considered the white surface total area (in mm^2^) and the mean thickness (in mm) of the cerebral cortex in the two hemispheres, thus obtaining a vector of 314 characteristics for each subject.

### Feature classification

We analyzed the brain image features with both standard two-class classifiers and OCC based on SVMs. The SVM (Vapnik, 1995) are quite extensively applied as conventional binary classification algorithms. They are supervised binary classifiers that require a training set of labeled input examples to learn the differences between the two sample classes, and a labeled test set to quantify the classification performance.

In the context of classification of brain images, each input example is a vector ***x*** of features extracted from each input image. The label **y** associated to each input example indicates its membership, e.g., “1” for vectors belonging to the patients class, “−1” for controls. Detailed information about two-class SVM can be found in (Pontil and Verri, 1997; Ben-Hur and Weston, 2010). Basically, during the training phase an optimization problem is solved to identify the largest-margin hyperplane (**w**·***x*** + b = 0 for linear kernel SVM) allowing for an optimal separation of the training examples of the two classes. The goal is to find a vector **w** and a scalar b, which maximize the margin, i.e., the distance between the two classes in the direction of **w**.

The SVM can then predict the classification of an unlabeled input vector by checking on which side of the separating hyperplane the example lies.

The SVM belong to the class of kernel methods (Schölkopf and Smola, 2002; Shawe-Taylor and Cristianini, 2004), i.e., they depend on data only through dot products. To achieve good separation results even in case of non-linearly separable classes, the dot product can be replaced by a kernel function, which computes a dot product in some (possibly) higher dimensional feature space. This allows carrying out a linear classification in the feature space, without explicitly mapping in it the original observations. The separating hyperplane found in the feature space corresponds to a non-linear boundary in the input space. This property is commonly known as kernel trick. Formally, a kernel function is defined as a function that, given two observations ***x***, ***x***′ ∈ *X*, satisfies *k*(***x***,***x***′) = ϕ (***x***) ϕ′(***x***′), where *X* is the input space or domain and ϕ is a function mapping from *X* to a feature space. In this case, the prediction of the class membership of an unlabeled input vector is performed by mapping it into the feature space, and checking on which side of the separating hyperplane the example lies.

Among the non-linear kernel functions the Radial Basis Function (RBF) kernel is the most popular. It depends on the Euclidean distance between the examples and it is defined as *k*(**x, x'**) = exp(−γ ||**x - x'**||^2^). The parameter γ determines the smoothness of the boundary (in the input space). Like the regularization parameter C in linear-kernel SVM, also the parameter γ is usually set using heuristics or tuned using cross-validation procedures.

Taking inspiration from the two-class SVM, Tax and Duin (2004) addressed the OCC problem by proposing a method to obtain a spherically shaped boundary around the target set. The method is called Support Vector Data Description (SVDD). During the training phase, an optimization problem is solved to minimize the volume of the sphere by minimizing the square of its radius, while demanding that the sphere contains most of the training objects. Similarly to what happens in two-class SVM, a regularization parameter C has to be set to control the trade-off between the sphere volume and the errors allowed in the target set.

Schölkopf et al. (2000) in presented a new formulation of two-class SVM, where the C parameter was removed and replaced with a new parameter ν with a more natural interpretation: it is an upper bound to the fraction of misclassifications and margin errors and a lower bound on the fraction of support vectors (SV). The authors showed that for certain parameter settings, the results of this new algorithm coincide with the conventional one. Moreover, desirable properties of previous SV algorithms are retained.

In 2001, Schölkopf and colleagues modified the previous approach (Schölkopf et al., 2000) to address the OCC problem and called the new algorithm single-class SVM. During the training phase of a single-class SVM, a hyperplane is placed such that it separates the target set from the origin with maximal margin. Similarly to the standard two-class SVM, when a more flexible data description is required, an implicit mapping of the data into another (possibly high dimensional) feature space is defined, such that the dot product in this feature space can be computed by evaluating a simple kernel function. An ideal kernel function would map the target examples onto a bounded, spherically shaped area in the feature space and outlier objects outside this area.

The constrained optimization problem to be solved is formulated as follows:

(1) Minimize(w,ξ,ρ)………12||w||2+1νN∑i = 1Nξi−ρ

subject to

(2) $$\begin{array}{l} \text{w}\;\phi \left({\text{x}}_{\text{i}}\right)\geq \rho -\xi_{\text{i}};\text{ i}=1,\ldots ,\text{N};\;\xi_{\text{i}}\geq 0. \end{array}$$

where ***x***_*i*_ are the target examples. The single-class SVM attributes a new point **x** to the target or the outlier class by evaluating which side of the hyperplane it falls on in the feature space.

As in two-class algorithm, the regularization parameter ν **∈** (0, 1] has to be set. Similarly to what happens in the two-class case, it can be interpreted as an upper bound on the fraction of training points outside the estimated region, and a lower bound on the fraction of support vectors. It is usually set based on its meaning or tuned using cross-validation procedures.

Although the method by Schölkopf et al. (2001) does not find a closed boundary around the data, it gives comparable solutions to Tax and Duin approach when the data is preprocessed to have unit norm (Tax and Duin, 2004). In particular, this happens when a RBF kernel is used. More in details, the solutions of the two approaches are identical when the same RBF kernel with γ and *C* = 1/(ν*N*), where *N* is the number of objects in the target set, are used, as reported by Sato et al. (2012a). In their practical implementation the two approaches operate comparably. Both perform best when the RBF kernel is used. Like in the two-class situation, the parameter γ can be set using heuristics or tuned using cross-validation procedures.

**Figure 1:**
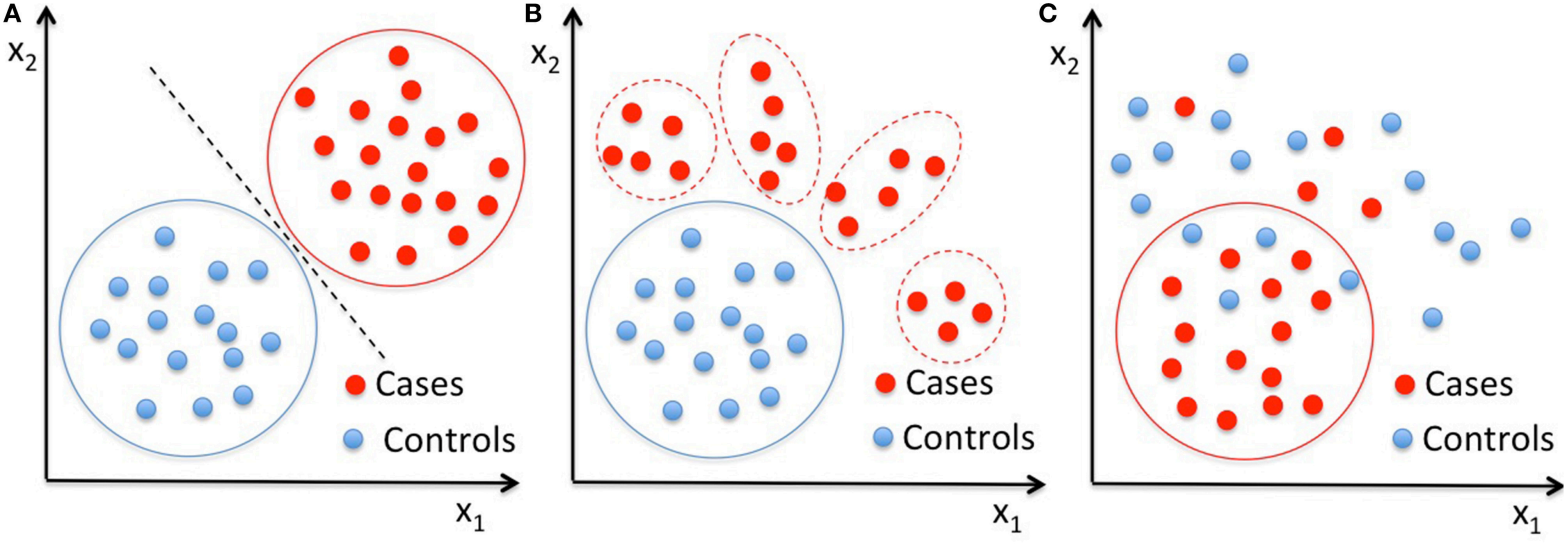
**Pictorial data representation in a two-dimensional space. (A)** Classical application of a binary classifier to distinguish two well-characterized groups of subjects; **(B)** OCC approach based on the hypothesis that the control group is the homogeneous baseline in comparison to which, subjects with different diseases cluster out of the boundary enclosing the controls; **(C)** schematization of the OCC result in the case where common features associate the patients, thus the OCC boundary encloses most patients leaving outside most control cases. Solid lines are drawn around the groups entering the training of the classifiers, i.e., both case and control subjects are needed in training a binary classifier **(A)**, whereas only on control cases in **(B)** and only the patients' group in **(C)** are necessary to train a OCC, respectively.

In this work, we first applied two-class SVM to the vector of 314 characteristics extracted for each subject of our datasets to obtain reference classification performances, and then we analyzed the same data with single-class SVM with RBF kernel. We performed the classification on the male subset and on the female subset separately, and then on the entire dataset. As pictorially shown in Figure 1, in contrast to the standard two-class classification approach to distinguish two well-characterized groups of subjects (Figure 1A), the OCC based on SVM can be implemented to make a multivariate description of the normative data, and thus to define a homogeneous baseline in comparison to which subjects with different diseases cluster out of the boundary enclosing the control group (Figure 1B). However, the latter approach relies on the hypothesis that the control group consists of typical individuals sharing a core of common distinctive features at neuroanatomical of functional levels, whereas non-typical individuals present alterations in many different ways, especially when highly heterogeneous condition are investigated, such as the ASD. Thus, in case the control class is not extremely homogeneous and it is matched to the patient class for many known aspects (e.g., age, gender, NVIQ) except for the presence of the disease condition, it may happen that OCC based on SVM capture common features according to which the patients can be described (Figure 1C). In the latter case, a boundary enclosing most patients can be identified, according to which most controls lie outside.

### Pre-image for RBF kernel

In the linear kernel classifiers, the entries of the vector **w** can be directly considered as the relative weights of each characteristic for the decision function (Gori et al., 2015). Conversely, in the non-linear case (e.g., the RBF kernel), the interpretation of the vector **w** is non-intuitive and complex, since the separating hyperplane is found in the feature space. Since the map Φ is non-linear, we cannot generally assert that each vector **w** in the feature space will have a *preimage* under Φ, i.e., a point ***z*** in the input space such that Φ(***z***) = **w**. In the present work we used for the single-class SVM with RBF kernel the approach proposed in by Schölkopf et al. (1999) to generate *preimages* by approximating the inverse mapping from the feature space to the input space.

Additionally, to understand which features and which neuroanatomical regions drive the SVM boundary definition, we tailored the permutation testing method (Mourão-Miranda et al., 2005; Wanh et al., 2007; Gaonkar and Davatzikos, 2013; Gori et al., 2015) to the case of single-class classifiers.

## Results

### Classification performance

The Freesurfer pipeline was applied to preprocess the MRI of each subject according to the Freesurfer guidelines.

Patients with ASD and controls were matched on age and NVIQ, thus resulting in 20 matched case-control pairs in the female subset and in 19 matched case-control pairs and one group constituted by two patients and one control in the male subset, because of its dimensionality. We used these *M* (*M* = 20 for male and female subsets considered separately, *M* = 40 for the entire dataset) matched groups as subsets in a cross-validation procedure to evaluate the performance of the SVM classifiers: for each subset *S*_*j*_, *j* = 1,…,M, we trained the SVM using the remaining subsets *S*_*i*_, *i* ≠ *j*, while retaining *S*_*j*_ as testing subset. Then we repeated the training and testing process for each subset, thus obtaining an estimate of the classifier performance on the entire dataset. Given the nature of the subsets, we refer to the cross-validation procedure we applied as leave-pair-out cross-validation (LPO-CV).

The performance of the SVM classifiers is evaluated in terms of the sensitivity (percentage of subjects with disease correctly identified, i.e., true positive rate) and the specificity (percentage of control subjects correctly identified, i.e., true negative rate). By varying the classifier decisional threshold, the trade-off between the sensitivity and the rate of false-positive detection can be represented in a curve known as Receiver Operating Characteristic (ROC) curve (Metz, 2006). The area under the ROC curve (AUC) is a global index to compare the ROC curves of different classifiers (Hanley and McNeil, 1982).

The LPO-CV scheme was implemented in the performance evaluation of both the two-class and the OCC SVM. A difference in the case of OCC SVM occurs only in the training step: to apply LPO-CV in the context of single-class classification we simply trained the single-class classifiers only on one class (target class) inside the cross validation, but tested it on both classes for the subset that was left out for testing.

To train and test two-class and single-class classifiers we used RapidMiner (http://rapidminer.com/) advanced analytics platform version 5.3, which includes both two-class and single-class SVM as a part of the LibSVM operator.

#### Two-class classification performance

A standard two-class SVM classification with linear and RBF kernels was carried out to discriminate subjects with ASD from controls, including the optimization of the SVM free parameters during the training. We reported the results in Table 2. The best performances we obtained were: AUC = 0.74 by using the linear kernel for the male subset and AUC = 0.65 by using the RBF kernel for the female subset. The reported two-class SVM performance already take into account the regularization and kernel parameter optimization, carried out through the nested LPO-CV procedure. We obtained the best results with linear kernel for males and with RBF kernel for females. These results represent a reference classification performance to compare with the new OCC SVM approach we propose.

**Table 2:** **The performances obtained in the ASD vs. CTRL classification with the two-class SVM, the OCC-SVM trained on the CTRL class, and the OCC-SVM trained on the ASD class are reported**.

**Two-class SVM**				**Male sample**		**Female sample**	
**Training set**	**Test set**	**Training specification**		**AUC**		**AUC**	
ASD and CTRL	ASD and CTRL	linear-kernel SVM for males/RBF for females	optimized C/optimized ν, γ	0.74		0.65	
	ASD and CTRL with NVIQ < 70			0.73		0.63	
	ASD and CTRL with NVIQ ≥ 70			0.8		0.63	
**One-class SVM**				**Male sample**		**Female sample**	
**Training set**	**Test set**	**Training specification**		**AUC**		**AUC**	
CTRL	ASD and CTRL	RBF	optimized ν, γ	0.50		0.50	
**One-class SVM**				**Male sample**		**Female sample**	
**Training set**	**Test set**	**Training specification**		**AUC**	***p*****-value**	**AUC**	***p*****-value**
ASD	ASD and CTRL	RBF	optimized ν, γ	0.74	0.012	0.68	0.016
	ASD and CTRL with NVIQ < 70			0.64	0.19	0.65	0.05
	ASD and CTRL with NVIQ ≥ 70			0.81	0.016	0.72	0.05
**One-class SVM**				**Male and female sample**			
**Training set**	**Test set**	**Training specification**		**AUC**				
ASD male and females	ASD and CTRL males and females	RBF	optimized ν, γ	0.64				
	ASD and CTRL males and females with NVIQ < 70			0.63				
	ASD and CTRL males and females with NVIQ ≥ 70			0.65				

*All classification performances are obtained in a leave-pair-out cross validation scheme, where the classifier training parameters are optimized within nested loops. The performances of the classifiers were separately evaluated in most cases on the subsets of ASD and CTRL subjects with low and high NVIQ values. The permutation test was also performed to assign a statistical significance p-value to the AUC in the case of OCC classification of male and female samples. ASD, Subjects with Autism Spectrum Disorders; CL, chance level; CTRL, control sample; NVIQ, non-verbal intelligence quotient; RBF, Radial Basis Function; SVM, Support Vector Machine*.

#### One-class classification performance

We first performed single-class classification by setting ν = 0.1 and γ using heuristics (i.e., as the inverse of the number of features). Then, following the procedure adopted in Mourão-Miranda et al. (2011), we carried out the optimization of the parameters ν and γ, within nested LPO-CV loops, as follows: at each iteration *j* of the outer LPO-CV scheme we had at disposal a subset ***A***_**j**_ = {***S***_*i*_}_*i* = 1, …, **N*, *i**≠**j**_ for training. Before training, we performed an optimization procedure based on an internal LPO-CV twice. In the first run, we kept the parameter ν fixed at an initial value (0.1) and we performed a LPO-CV for several values of γ in order to find its optimal estimate (i.e., the one that maximizes the AUC). To cover a wide range of possible γ-values, we let this parameter vary first on a coarse grid (19 steps from 2^−15^ to 2^3^ by power of 2); then, we refined the search in the interesting regions we identified. In the second run, we kept γ fixed at the optimal value and we performed a second optimization procedure to choose the optimal ν, using a coarse optimization with 10 linearly spaced values in the [0.01–0.5] range, then a fine search in [0.01–0.3] range using 20 linearly spaced values. A single-class SVM was finally trained on ***A***_*j*_ using the optimal values of γ and ν and tested on the subset ***S***_*j*_ left out in the outer LPO-CV scheme. This procedure was repeated for each j, each time leaving a different subset outside as test subset.

The intuitive approach for transforming a binary discrimination problem into a single-class task in the context of highly heterogeneous conditions like ASD is to use the control class as target class, figuring that it could enable the definition of a robust boundary, in relation to which the ASD patients would be classified as outliers. Consequently, we first trained a single-class SVM by considering only control examples to form the decision boundary, thus discarding information about the ASD class during the training phase.

This would be the optimal approach if the control class had characteristics of homogeneity, since the single-class SVM could capture the control class structure, by adjusting itself to its properties. This would allow recognizing ASD examples as outliers, even if the available ASD samples were not representative of the real ASD population, due to the extreme ASD heterogeneity.

However, the results obtained in this case in terms of AUC were not above the chance level, despite the optimization on of the parameters ν and γ.

Therefore, we repeated the same procedure using the ASD patient group as the target class to investigate whether there was a consistent neuroanatomical pattern among the ASD patients in relation to which the controls can be classified as outliers.

The results obtained in terms of AUC were: AUC = 0.73 for the male subset and AUC = 0.66 for the female subset by setting ν = 0.1 and γ using heuristics; by optimizing the parameters ν and γ, AUC = 0.74 for the male subset and AUC = 0.68 for the female subset. We summarized in Table 2 the classification results we obtained in the different classification experiments we conducted.

These results show that the control class does not have characteristics of homogeneity allowing recognizing ASD examples as outliers. Conversely, there is a common structure among the ASD patients that the OCC–SVM could capture. The OCC–SVM assigns to a test case a continuous output providing the confidence for it to belong to the target class or to be an outlier. To investigate whether the AUC performance obtained in the case-control discrimination were significantly above the chance level we implemented the permutation test. To obtain the null distribution of the AUC, we carried out a permutation test procedure in the training phase, tailoring it to the single-class classifiers. In a standard two-class classification the permutation test is performed by randomly exchanging several times the class labels (i.e., by randomly assigning positive and negative labels either to the ASD and the control cases) and repeating the classification procedure. In the OCC case, the training is performed on the target class only, e.g., the ASD class in our analysis. To find the null distribution for AUC, we permuted the group labels 10000 times, creating “artificial” training datasets where randomly chosen ASD cases were exchanged with their matched controls. Each “artificial” ASD dataset was then used to train one single-class SVM, i.e., to generate a decision function. We counted the number of times the AUC exceeded the value obtained with the real class labels. Dividing this value by the number of permutations provided a statistical significance for the AUC. The permutation testing procedure was applied separately for the male and the female subsets. We used the Matlab (The MathWorks, Inc.) interface to the LIBSVM package (http://www.csie.ntu.edu.tw/~cjlin/libsvm/) to train the single-class classifiers in the permutation test procedure, implementing the RBF parameter optimization as nested LPO-CV loops. The AUC values and the significance values of the permutation test are reported in Table 2.

Finally, to investigate the impact of some known heterogeneity factors present in the ASD sample, we estimated the OCC performance on the entire dataset, by using the group of both male and female subjects with ASD as the target class. We achieved AUC = 0.64 in the case-control discrimination. This slight performance decrease is not surprising and we ascribed it to the introduction of the gender as additional heterogeneity factor.

Another relevant heterogeneity factor in our data is the NVIQ of subjects, which is in the [31–123] range. If the performance of OCC trained on male subjects with ASD (leading to AUC = 0.74 on the male population) is evaluated separately on the subsamples of subjects with NVIQ ≥ 70 and NVIQ < 70, the values of AUC = 0.81 and AUC = 0.64 were obtained, respectively. A similar trend holds for the OCC trained on the female subjects with ASD (leading on AUC = 0.68 on the female population). In this case AUC = 0.72 and AUC = 0.65 were obtained on the subsamples of subjects with NVIQ ≥ 70 and NVIQ < 70, respectively.

### Maps of discriminant brain regions

To understand which of the 314 characteristics (i.e., which brain regions and which of the five computed features) are the most relevant to the single-class SVM boundary definition, we trained a single-class SVM with RBF kernel using all the ASD patient group as the target class (with ν = 0.1 and heuristic γ) and we applied the algorithm proposed in Schölkopf et al. (1999) to generate the *preimage* vector ***z***. We used the Statistical Pattern Recognition Toolbox for Matlab (STPRTool) (http://cmp.felk.cvut.cz/cmp/software/stprtool/index.html) to generate the *preimage*.

To obtain the null distribution of the preimage ***z***, we carried out a permutation test in the training phase by permuting the group labels 10000 times, as described above. Each “artificial” ASD dataset generated a decision function and a corresponding preimage vector ***z***. Since each component of ***z*** corresponds to one of the 314 characteristics, we obtained a null distribution associated with every characteristic. We counted the number of times the so-generated components of ***z*** exceeded (comparing the absolute values) the corresponding values in the ***z*** map obtained for the real ASD cases; dividing this value by the number of permutations provided a statistical significance map. Then, we retained only the characteristics with *p* < 0.05. The relevant characteristics (features and regions) resulting from the permutation test applied to male and female data groups are shown in Tables 3, 4. As stated before, in case of linear-kernel SVM, the separating hyperplane **w** can be represented as an image showing which brain regional features are more relevant for the classification problem. Additionally, the sign of each element of **w** directly identifies whether the corresponding feature is greater either in the case or in the control group. This information is inaccessible when using RBF, as the linear problem is solved in the space defined by the non-linear transformation **w** = Φ(***z***), thus the signs of the elements of the *z* vector (i.e., the *preimage*) do not indicate whether a relevant feature in the classification problem is greater in the case or in the control group. To foresee this information, which is important to compare the result we obtain with those presented in other studies, we simply analyzed the distributions of each feature and reported the trend of the sign of the case-control difference. We indicate in Tables 3, 4 with arrows pointing up/down the features significantly contributing to the OCC boundary definition whose individual trend is toward increased/decreased values in the group of male subjects with ASD with respect to matched controls.

**Table 3:** **Relevant brain regions and features for the male group (***p*** < 0.05)**.

**Male subset**		**Feature**				
**Hemisphere**	**Region**	**Area**	**Mean curvature**	**Thickness**	**Thickness standard deviation**	**Volume**
lh	Medial orbitofrontal cortex		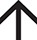			
	Middle temporal gyrus	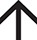				
	Pars triangularis	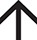	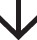			
	Posterior cingulate cortex	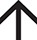				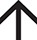
	Transverse temporal gyrus	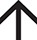				
rh	Insula	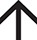	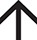			
	Medial orbitofrontal cortex		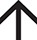			
	Pars opercularis	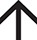				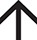

*Arrows pointing up/down indicate the features significantly contributing to the OCC boundary definition, whose individual trend is toward increased/decreased values in the group of male subjects with ASD with respect to matched controls*.

**Table 4:** **Relevant brain regions and features for the female group (***p*** < 0.05)**.

**Female subset**		**Feature**				
**Hemisphere**	**Region**	**Area**	**Mean curvature**	**Thickness**	**Thickness standard deviation**	**Volume**
lh	Caudal anterior cingulate cortex			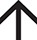		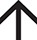
	Cuneus	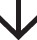				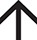
	Enthorinal cortex				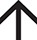	
	Inferior temporal lobe	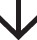				
	Lateral orbitofrontal cortex				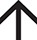	
	Pars opercularis			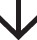		
	Posterior cingulate				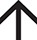	
	Precuneus	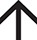				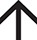
	Rostral anterior cingulate cortex		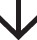			
	Transverse temporal gyrus					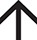
rh	Caudal middle frontal gyrus	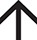				
	Cuneus				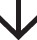	
	Enthorinal cortex				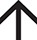	
	Pars opercularis		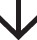			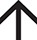
	Pars triangularis			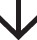	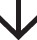	
	Postcentral gyrus	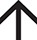				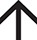
	Precuneus	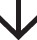				
	Rostral anterior cingulate cortex		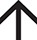			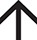
	Superior parietal cortex		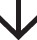			
	Superior temporal gyrus		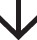			

*Arrows pointing up/down indicate the features significantly contributing to the OCC boundary definition, whose individual trend is toward increased/decreased values in the group of female subjects with ASD with respect to matched controls*.

**Figure 2:**
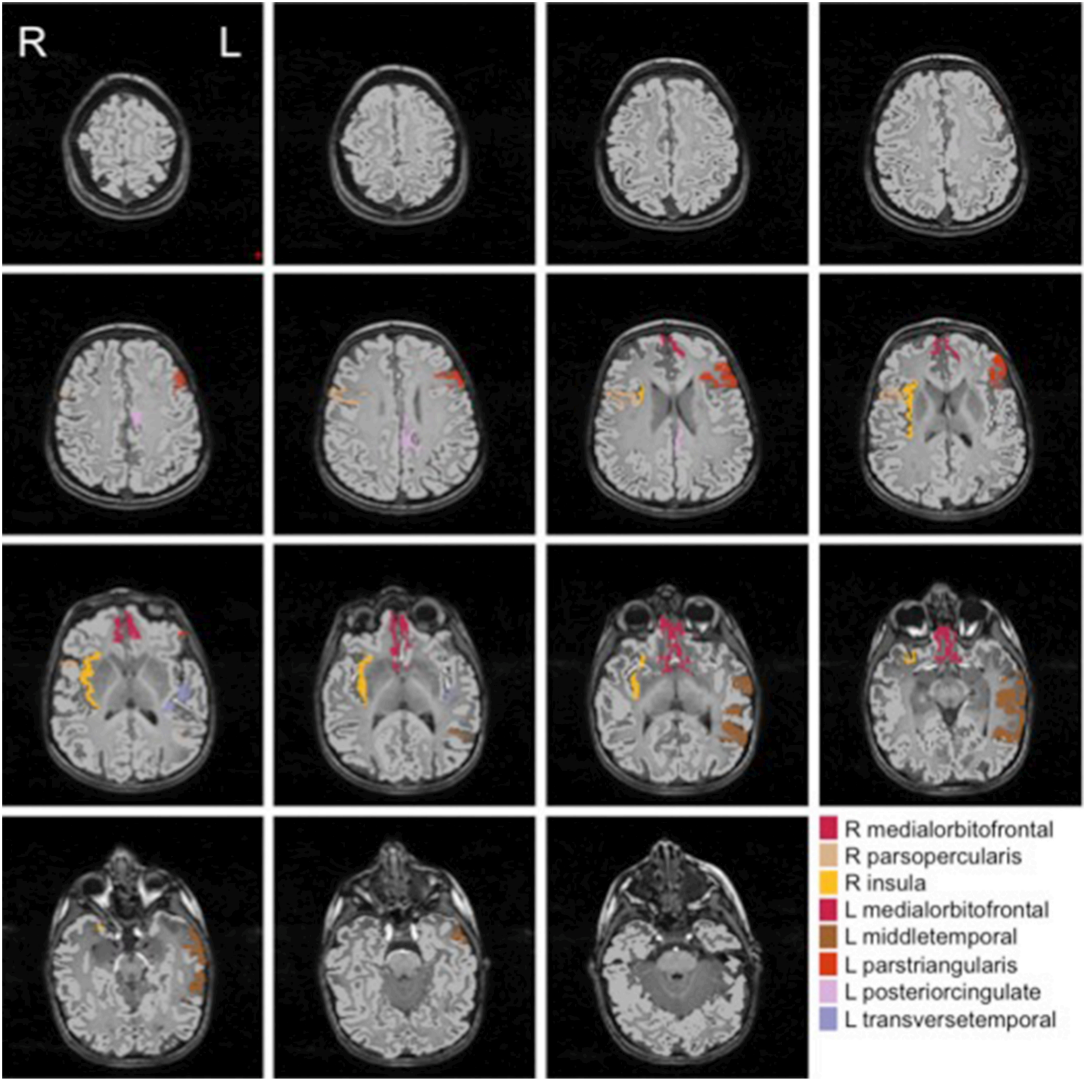
**Brain regions most contributing to the definition of the OCC boundary for the male group**.

**Figure 3:**
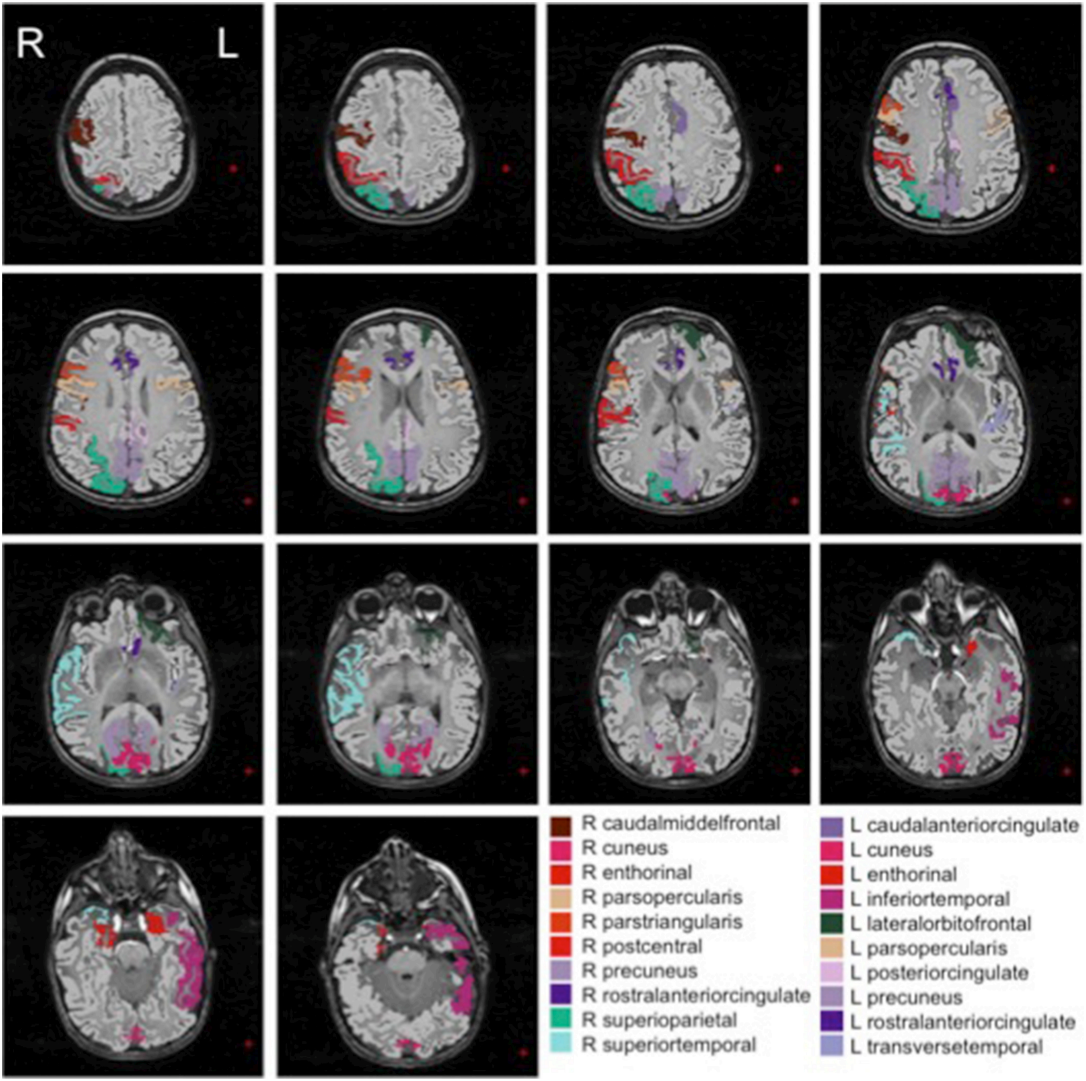
**Brain regions most contributing to the definition of the OCC boundary for the female group**.

We show in Figures 2, 3 the brain regions contributing most to the definition of the OCC boundary, as reported in Tables 3, 4 for male and female subsets, respectively. For the male population the main regions are: left (L) and right (R) medial orbito frontal cortices, L pars triangularis and R pars opercularis of the inferior frontal gyrus, middle temporal cortex and R insula. For the female population the main regions are: L and R caudate anterior cingulate, pars opercularis, posterior cingulated, cuneus; R pars triangularis postcentral gyrus, superior temporal cortex and superior parietal cortex. They are mostly among the network of structural brain alterations widely reported in the population with ASD, including frontal and temporal areas. Thus, despite the phenotypical heterogeneity in ASD, a common neuroanatomical profile of core features could be detected with the OCC SVM approach.

## Discussion

We analyzed the brain structural MRI features of patients with ASD with OCC SVM, starting with the estimation of the OCC performance in the ASD vs. controls discrimination task. Then, we investigated whether the distribution of patterns of brain structures in control subjects is homogeneous enough to enable the definition of a robust boundary, in relation to which the patients with ASD would be classified as outliers. This approach is consistent with previously proposed methods where OCC were implemented on fMRI features to build multivariate normative rules on the healthy control population, which would allow recognizing abnormal cases as outliers (Mourão-Miranda et al., 2011; Sato et al., 2012a,b). In the specific case of the population of young children with ASD we analyzed, we found out that an OCC boundary enclosing the controls, built upon structural MRI brain features, will allow most ASD cases to fall within the boundary. By contrast, a consistent pattern among the patients with ASD could be identified by the OCC approach, which provided a boundary in relation to which most controls were classified as outliers. In other words, we found out evidence that the control group is the more heterogeneous one and therefore the hypersphere or decision boundary enclosing most of the controls contains data in the ASD range. Vice versa, the ASD group showed a common structure that the SVM OCC could capture. This apparently counterintuitive result might be understood in the light of the following considerations. First, we performed a priori heterogeneity reduction of the ASD sample by excluding subjects with ASD secondary to known causes and/or with dysmorphic features (see Exclusion criteria in Section Participants and MRI Data Acquisition). Conversely, the control group was highly heterogeneous, since it comprises children who span the full range of cognitive ability (NVIQ score range: 31–123). This selection is motivated by the our primary choice of including within the cases all ASD children who performed MRI, since focusing just on those who are high-functioning would be non-representative of ASD population comprising about 55% of subjects in the intellectual disability range (Charman et al., 2011). As a necessary consequence, subjects with idiopathic developmental delay (DD) were included within the control group in order to obtain a match on NVIQ and thus a reliable MRI data interpretation (Crone et al., 2010). Therefore, the heterogeneity of MRI structural features within the control sample could be ascribed not only to the normal inter-individual brain variability that occurs among individuals with typical development (Wilke and Holland, 2003; Kanai and Rees, 2011), but also to the heterogeneous subsample of individuals with DD. In fact, by definition, idiopathic DD appears to be a highly heterogeneous disorder in terms of etiopathogenesis and clinical features: it is therefore plausible that also its neuroanatomical substrate is heterogeneous and hence contributes to amplify the cerebral differences detected in our control population. Moreover, we restricted our analysis to an early and relatively narrow age-range (2–6 years), a time-period in which structural MRI findings of ASD patients are more consistent and less heterogeneous across studies (Wolff and Piven, 2013). Specifically, a overgrowth of WM and GM before 2 years of age followed by a growth rate reduction that lead to brain volumes similar to typical children by approximately the school-age period was frequently reported (Lenroot and Yeung, 2013). In addition, by focusing on preschoolers only, we captured the pattern of brain alterations taking place near the clinical onset of the disorder and therefore we minimized the influence of different post-natal variables (e.g., environmental factors, psychiatric comorbidities, rehabilitative intervention) on brain structure. In other words, it is possible that a common altered brain presentation is more frequent in the early stage of the ASD disorder and that inter-subject variability in ASD populations progressively increases with age.

The present work is a proof of concept that the OCC framework can be applied to neuroimaging data to investigate if consistent patterns of alterations do exist even in heterogeneous populations such as ASD. Despite the results we found need to be confirmed against a larger population, the approach we present here is a preliminary step aiming to set up a strategy to identify common altered features in specific disorders.

Analogously, a common brain endophenotype in ASD individuals was detected with different methods of data acquisition, including electroencephalogram (EEG) spectral coherence (Duffy and Als, 2012), voxel based morphometry (Uddin et al., 2011), functional MRI (White et al., 2014), functional connectivity (Murdaugh et al., 2012), and diffusion tensor imaging (Ingalhalikar et al., 2011). These findings did not support the extreme variability of cerebral structure and function in ASD patients, as suggested by other investigations (Alexander et al., 2007; Nordahl et al., 2012; Hahamy et al., 2015).

Since we found the rather limited maximum AUC value of 0.74 for the male subset, it is important to highlight that among control subjects we included patients with DD that from a clinical point of view can frequently be considered in the ASD differential diagnosis. Therefore, it is possible that not only at the behavioral level, but also at the neuroanatomical level individuals with ASD and individuals with DD share some features that make the brain-based distinction between each other more difficult. This interpretation of our results is further supported by the fact that the AUC values reported on the subgroups of subjects with high NVIQ values are systematically higher than those obtained on the subgroups with low NVIQ values (see Table 2), whereas the performances obtained on the entire samples are positioned in between, as expected. It happens in the analysis of the male and the female subsamples and of the entire data sample. According to this view, the capability to differentiate participants into patient and control groups improved when we restricted the analysis to ASD patients with NVIQ ≥ 70 and therefore we included among control subjects only individuals without DD (AUC = 0.81 and AUC = 0.72 for female and male subsamples, respectively).

The features which had the highest discriminative ability between the cases (both males and females) and the controls belong to four cerebral regions -posterior cingulate cortex (PCC), pars opercularis, and pars triangularis of inferior frontal gyrus, transverse temporal gyrus- all of which would represent a neuroanatomical signature of pre-schoolers with ASD. Specifically, we detected increased left PCC volume in ASD patients. Notably, this region has been included in the default mode network as one of the highest baseline energy consuming regions (Raichle et al., 2001) and has been implicated in arousal and awareness (Vogt and Laureys, 2005), autobiographical memory retrieval (Maddock et al., 2001), as well as in cognitive flexibility and in the ability to regulate the breadth of attention (Leech and Sharp, 2014), functions frequently impaired in patients with ASD (Bruck et al., 2007; Leung and Zakzanis, 2014; Orekhova and Stroganova, 2014).

In addition, we identified a significant alteration of pars opercularis and pars triangularis in patients with ASD. These inferior frontal regions together comprise Broca's area, which is primarily involved in higher-order abilities such as expressive language, action imitation, attribution of mental states, and empathy (Iacoboni and Dapretto, 2006). Disruption of this area may therefore lead to core ASD symptoms (Dapretto et al., 2006). In particular, our finding of reduced cortical thickness (CT) in the pars opercularis is in line with results of Zielinski et al. (2014) in children and adolescents with ASD and of Hadjikhani et al. (2006), who reported a local decreases of CT in the pars opercularis of 14 high-functioning adults with ASD compared with matched control subjects. Also, decreased CT in the pars triangularis is in concordance with a previous study demonstrating focal patterns of cortical dysmaturation in children with ASD (Jiao et al., 2010). At the volumetric level, an increase in pars opercularis and pars triangularis was identified in children with ASD compared to controls (Knaus et al., 2009) while an opposite finding characterized adults with high-functioning ASD (Yamasaki et al., 2010), supporting an altered trajectory of neurodevelopment in the autistic disorder. Interestingly, a highly localized structural alteration consisting in a significant reduction of the sulcus maximum depth was recently detected in the Broca's area of young children with ASD (Brun et al., 2016), suggesting its possible role in facilitating early ASD identification.

Our individuals with ASD showed increased volumes (females) and area (males) of the left transverse temporal (Heschl's) gyrus relative to controls. The transverse gyrus of Heschl includes the primary auditory cortex and is critically implicated in early auditory processing (Galaburda and Sanides, 1980). Several ASD symptoms, including altered auditory responsiveness (O'Connor, 2012), language perception and acquisition, are strictly related to this cerebral region. Previous cross-sectional investigations on older patients with ASD failed to observe any volumetric alteration in Heschl's gyrus (Gage et al., 2009; Knaus et al., 2009): it is possible that neuroanatomical differences in this area are age-related, and therefore detectable in our sample of ASD preschoolers, but not more during childhood and adolescence.

Crucially, a longitudinal investigation reported a reduced growth of Heschl's gyrus white matter in the left hemisphere as well as in the right Heschl's gyrus gray matter of children with ASD (Prigge et al., 2013), supporting an abnormality in the trajectory of cerebral development in the ASD group.

It is worth mentioning that the most discriminative brain features that characterize young children with ASD in the current investigation are largely overlapping with those identified in a resting-state connectivity analysis of brain lateralization in 447 high-functioning individuals with ASD (Nielsen et al., 2014). Therefore, a consistent finding was detected in these two studies, despite they do present substantial differences not only as to the numerosity, IQ sample and imaging modalities, but also as to the age of participants (preschoolers in our study vs. individuals across a wide range of ages starting from 6 years in the paper by Nielsen and colleagues), and sites of MRI acquisition (single site in our study vs. multiple sites in Nielsen's report). Hence, this replicated result would open the door to speculation that, irrespective of demographic and clinical features, selective alteration in language and default mode areas is a universal cerebral endophenotype of ASD.

In conclusion, results from the present study suggest that a distinctive neuroanatomical profile could be identified in preschoolers with ASD, independently of their gender, age, and NVIQ. In fact, beside the well-known heterogeneity of the ASD condition, patients seem to share common neuroanatomical substrates that appear to comprise language and default mode regions and could represent the core brain alterations of the disorder in the preschool age.

Several limitations of the current work and directions for future studies should be highlighted. First, the classification performances obtained are quite modest and, in some specific cases, the performances of the OCC classifiers are not significantly different from the chance level, as it happens for example on the sub group of male subjects with low NVIQ values (see Table 2). More populated data samples would be necessary to understand whether with improved statistical sensitivity the two overlapping classes can be effectively disentangled. Second, the relatively limited sample size prevented us from reliably subgrouping ASD patients on the basis of gender and NVIQ for investigating possible brain correlates of phenotypic differences. Third, we did not implement in our classification model any feature selection technique. Due to the large number of features (314) with respect to the data sample size we are working with a high risk of overfitting the models. As we are interested most in the discrimination maps generated by the OCC than in the classification performance by itself, the overfitting problem does not seem a major issue. However, to investigate in depth the separability of the ASD and control samples, and to understand whether the modest AUC values we obtained on cross validation are due to the lack of generalization abilities due to model overfitting, a feature selection technique should be implemented. Finally, since patients with ASD were recruited from an ASD Unit in a large tertiary hospital and research university that evaluates patients under 18 years of age from all over Italy, we may not have been fully able to capture children at the less severe end of the spectrum.

Future investigations will involve: (i) analyzing whether a distinct clinical symptoms or behavior profile characterized the outliers within the ASD cohort; (ii) evaluating whether the brain ASD endophenotype detected in the first years of life remains stable over time, or vice-versa developmental changes in ASD symptom profiles impact also on brain structure; (iii) including analysis of patients with other neurodevelopmental disorders which display overlapping clinical features with ASD [e.g., language disorder, social (pragmatic) communication disorder, attention-deficit/hyperactivity disorder, stereotypic movement disorder] in order to verify the specificity of the discriminative brain pattern here identified in ASD patients.

## Author contributions

AR and SC designed the study; IG, AG, and AR carried out data processing and analysis; SC and FM interpreted the results; AR, IG, and SC drafted the manuscript; AG edited the manuscript; FM critically revised the manuscript for important intellectual content.

### Conflict of interest statement

The authors declare that the research was conducted in the absence of any commercial or financial relationships that could be construed as a potential conflict of interest.
